# Astrocytic Ror2-induced imbalance in brain and gut homeostasis contributes to chronic post-thoracotomy pain

**DOI:** 10.3389/fnagi.2025.1675725

**Published:** 2025-11-07

**Authors:** Chaoqun Liu, Le Shen, Li Xu, Afang Zhu, Yuguang Huang

**Affiliations:** 1 Department of Anesthesiology, Peking Union Medical College Hospital, Chinese Academy of Medical Sciences and Peking Union Medical College, Beijing, China; 2 Department of Anesthesiology, National Cancer Center/National Clinical Research Center for Cancer/Cancer Hospital, Chinese Academy of Medical Sciences and Peking Union Medical College, Beijing, China

**Keywords:** Ror2, astrocytes, CCL2, CXCL1, anterior cingulate cortex, short-chain fatty acids, chronic post-thoracotomy pain

## Abstract

**Background:**

Receptor tyrosine kinase-like orphan receptor 2 (Ror2) plays an indispensable role in mediating acute and chronic pain. We previously demonstrated the involvement of spinal cord astrocyte-derived Ror2 in chronic post-thoracotomy pain (CPTP). Here, we further investigated the roles of anterior cingulate cortex (ACC) Ror2 and gut microbiota in CPTP.

**Methods:**

A rat CPTP model was established. The mechanical withdraw threshold and cold allodynia were measured to evaluate pain behavior. Intrathecal injection of AAV2/9-GFAP-miR30-shRor2 was used to knock down in vivo Ror2 expression in ACC astrocytes. The expressions of Ror2, the polarisation of astrocytes, the synthesis of CCL2 and CXCL1 in the ACC, and the levels of gut short-chain fatty acids (SCFAs) before and after intervention were assessed by RT-PCR, western blot, double immunofluorescence staining and gas chromatography-tandem mass spectrometry (GC-MS).

**Results:**

We observed elevated astrocytic Ror2 levels in the ACC of male CPTP rats, with astrocytes predominantly polarized into the A1 phenotype, characterized by increased astrocytic CCL2 and CXCL1 secretion, and a concomitant reduction in gut-derived SCFAs. Knockdown of astrocytic Ror2 through intrathecal AAV2/9-GFAP-miR30-shRor2 administration significantly alleviated mechanical hyperalgesia and cold allodynia caused by thoracotomy, restored the ACC A1/A2 astrocytic balance, reduced its CCL2 and CXCL1 expression, and increased gut-derived SCFA production in CPTP rats.

**Conclusion:**

These findings suggest that the facilitation of CPTP development by Ror2 is associated with disruptions in the A1/A2 astrocytic balance, chemokine production in the ACC, and SCFA levels in the gut microbiota. Inactivation of astrocytic Ror2 may serve as a targeted treatment to restore the balance of reactive astrocytes and mitigate their pathogenic effects.

## 1 Introduction

As one of the most painful surgical procedures, thoracotomy frequently results in acute and chronic pain, which may lead to frailty, pneumonia, atelectasis, prolonged hospitalization, diminished life quality, poor prognosis, and other adverse effects (Tiippana et al., 2003). Chronic post-thoracotomy pain (CPTP) is defined as recurring or persisting pain for at least 2 months post-surgery (Merskey and Bogduk, 1994). The incidence of CPTP varies from 25% to 57%, with approximately 10% of patients experiencing severe frailty as a complication (d’Amours et al., 1998; Pain Supplements, 1986; Wildgaard et al., 2016). Although CPTP is linked to inflammatory pain, neuropathic pain, and visceral pain, its specific pathogenesis remains unclear (Marshall and McLaughlin, 2020). Therefore, elucidating the mechanisms underlying CPTP is critical to identifying new treatment strategies.

Historically, reactive astrocytes in CNS pathologies were often discussed within the framework of the A1/A2 paradigm, where A1 astrocytes were proposed to be neurotoxic and A2 astrocytes neuroprotective (Li et al., 2020; Raghavendra et al., 2004; Vega-Avelaira et al., 2007). However, this binary classification is now recognized as an oversimplification. Growing transcriptomic evidence reveals a vast spectrum of astrocyte reactive states that cannot be encompassed by these two categories. While we reference the A1/A2 nomenclature due to its historical relevance in the field, our study describes the astrocytic responses in chronic pain in terms of their specific molecular signatures and functional alterations, which extend beyond this classical dichotomy. We recently discovered that A1/A2 transitions in spinal cord astrocytes are involved in CPTP progression (Liu et al., 2022). Moreover, in addition to the spinal cord, several brain regions, notably the anterior cingulate cortex (ACC) and insular cortex, also participate in chronic pain (Apkarian et al., 2005). We observed reactive A1 astrocytes in the cortex and hippocampus during CPTP (Zhu et al., 2023). The astrocytes reactivity in the ACC is associated with neuropathic and inflammatory pain; inhibiting this activation alleviates anxiety in chronic pain (Ikeda et al., 2013; Sun et al., 2016; Yamashita et al., 2014). These findings suggest that balancing A1/A2 astrocytes in the ACC may influence chronic postsurgical pain, offering a potential therapeutic target.

Emerging evidence highlights receptor tyrosine kinase-like orphan receptor 2 (Ror2) as a key regulatory molecule in the pathophysiological mechanisms of acute and chronic pain (Dajczman et al., 1991; Shi et al., 2012). We previously showed that both Wnt5a and its transmembrane receptor, Ror2, were markedly elevated in the dorsal root ganglion of CPTP rats (Zhu et al., 2018). Furthermore, Ror2 knockdown in astrocytes alleviated thoracotomy-induced chronic pain by re-balancing A1 and A2 astrocytes activity in the spinal cord (Liu et al., 2022). In addition, we observed reactive A1 astrocytes in the cortex during CPTP (Zhu et al., 2023), suggesting that Ror2 may mediate CPTP development by regulating ACC astrocyte phenotypes.

Neuroinflammation, characterized by the activation of astrocytes and microglia, is crucial in pain pathogenesis. Glial cell-derived chemokines are major contributors to this process (Kiguchi et al., 2012; Koyama et al., 2007). Increasing evidence suggests that chemokines, such as CCL2 and CXCL1, play a role in the development of chronic pain following nerve damage (Abbadie et al., 2009; Gao and Ji, 2010). CCL2 can bind to CCR2 or CCR4 receptors, activating peripheral nociceptive neurons and causing mechanical and thermal hyperalgesia (Bégin-Lavallée et al., 2016). CXCL1 is a key chemokine involved in immune responses. Zhang et al. (2013) found that intrathecal injection of CXCL1 induces hyperalgesia by activating spinal cord neurons in a spinal nerve ligation model, whereas CXCL1-neutralizing antibody counteracts this effect. These findings indicate that astrocytic CCL2 and CXCL1 contribute to chronic pain; however, their roles in the ACC neuroinflammation during CPTP development remain unclear.

The gut microbiota regulates brain behavior by modulating the gut–brain axis in neuropsychiatric diseases (Góralczyk-Bińkowska et al., 2022). Gut microbiota dysbiosis may lead to mental disorders, which, in turn, affect the gut microbiota composition, its metabolites, and gastrointestinal functions. Short-chain fatty acids (SCFAs), the primary gut microbiome-derived metabolites, comprise acetic, propionic, and butyric acids (Cryan et al., 2019). SCFAs are involved in chronic pain progression via the gut–brain axis (Ma et al., 2020; Zhou et al., 2021). However, it is unknown whether decreasing neuroinflammation by regulating astrocyte subtypes ameliorates pain-induced gut microbiota dysbiosis.

We adopted a rat CPTP model in this study to investigate the contribution of Ror2 to the polarization of A1/A2 astrocytes, its involvement in the ACC neuroinflammation, and its effects on SCFA production. We aimed to modulate the balance of reactive astrocytes in the ACC during the initial phase of pain and examine its effects on the corresponding mechanisms in the brain and gut.

## 2 Materials and methods

### 2.1 Experimental animals

The study used adult male Sprague–Dawley rats (6 weeks old, weighing 120–150 g) obtained from the Experimental Animal Centre of the Chinese Academy of Medical Sciences, Beijing, China. They were maintained under a controlled environment at 23 ± 1 °C, with a 12-h light–dark cycle, and had *ad libitum* access to food and water throughout the study. The Institutional Animal Care and Use Committee of the Chinese Academy of Medical Sciences reviewed and approved all experimental protocols (Approval No. XHDW-2019-00), which followed the guidelines outlined in the National Institutes of Health Guide for the Care and Use of Laboratory Animals. The rats underwent a 5–7-day acclimation period prior to the experiments. The animals were then randomly allocated to experimental or control groups, with at least 10 rats per group for pain behavior assessments and 3–6 rats for other experiments.

### 2.2 CPTP model

Prior to use, the instruments were autoclaved for sterilization. In accordance with Buvanendron (Buvanendran et al., 2004), a thoracotomy procedure was performed on rats anesthetized with 60 mg/kg pentobarbital sodium via intraperitoneal injection. The rats were mechanically ventilated using a volume-based mechanical ventilator (Harvard Inspira, Harvard Apparatus, MA, USA) designed for small animals after intubation with a 16-gauge catheter. A 3-cm incision along the right fourth intercostal space was made to reveal the intercostal muscles. Subsequently, a 1.5-cm incision was carefully made through the intercostal muscle and pleura, just above the superior margin of the fifth rib, ensuring that the lung was not harmed. A micro retractor with blunt teeth was then gently inserted and secured in position, keeping the fourth and fifth thoracic rib spaces separated by 8-mm for 1 h. The surgical site was kept moist with damp gauze. To remove air from the chest, a 22-gauge venipuncture cannula was inserted, and the air was aspirated using a 5-mL syringe before closure. Layer-by-layer suturing was performed on the ribs, muscles, and skin. The intubation catheter was removed once spontaneous respiration resumed. Rats in the sham group were subjected to skin and pleural incisions, but no rib retraction was performed.

### 2.3 Behavioral assessment

Behavioral assessments were conducted daily in a blinded manner and at consistent times. Before testing in a quiet room, each rat was confined individually in a loosely restrained cage (8 × 9 × 20 cm) for half an hour. To assess mechanical hyperalgesia, the mechanical withdrawal threshold (MWT) was measured using an electronic von Frey device (IITC Life Science Inc., Woodland Hills, CA, USA). Force was applied perpendicularly to the dorsal skin near the surgical site, increasing stepwise until a response occurred. Positive reactions were defined as behaviors, such as rotating the trunk 180°, scratching the right upper dorsal skin with the hind paw, or exhibiting signs of shivering or distress vocalization (Chi-Fei Wang et al., 2013). Hyperalgesia was defined as an MWT ≤4 g, determined by averaging three successful recordings with intervals exceeding 30 s (Buvanendran et al., 2004).

Cold allodynia was evaluated by observing the behavioral response of rats to cold acetone application. Briefly, a 0.5-mL volume of cold acetone was gently applied to an area 2 cm proximal to the wound site. Within 1 min of application, the number of scratches on the hind paw and the rotational movement frequency toward the acetone-treated area were recorded to quantify the response. The final assessment was based on the average of three measurements taken at intervals of over 5 min. Rats scratching three or more times within 1 min were considered to have cold allodynia (Buvanendran et al., 2004).

Behavioral assessments were performed before and at various intervals after thoracotomy on days 1, 3, 5, 7, 10, 14, 18, and 21. Behavioral assessments were also conducted to investigate the effects of intrathecal AAV2/9-GFAP-miR30-shRor2 injections on pain, with testing performed at baseline (day before injection) and at the same intervals post-injection.

### 2.4 Intrathecal administration

Intrathecal administration of adeno-associated virus (AAV) genes enables efficient central nervous system targeting (Bey et al., 2017). To suppress Ror2 protein expression in the ACC astrocytes, two siRNAs (siRNA1, with the sequences 5′-CAGCCAGCACAAACAGGCCAAACTT-3′, and siRNA2, with the sequences 5′-GAGGCCGTCATGTACGGAAAGTTCT-3′) targeting rat ACC astrocytic Ror2 were synthesized. These sequences were incorporated into the Mir30 context and packaged into an AAV vector (Hanbio Technology, Shanghai, China). To ensure that the AAV had sufficient time to be effective, AAV2/9-GFAP-miR30-shRor2 and the control vector, AAV2/9-GFAP-Control, were administered intrathecally 21 days before thoracotomy.

During the injection procedure, anesthetized rats were prone-positioned, and a dorsal midline incision was performed to make the L4–L5 intervertebral space visible. While stabilizing the L5 spinal process with the left hand, the L4–L5 disk space was carefully punctured with a 50-μL microsyringe needle held perpendicularly with the right hand, thereby ensuring successful penetration through the ligamentum flavum. Once a tail flick was detected, micro-contraction was discontinued, confirming the proper placement of the intrathecal needle.

Subsequently, slow intrathecal injection with AAV2/9-GFAP-miR30-shRor2 or AAV2/9-GFAP-Control (both 40 μL) was performed, with the needle maintained in place briefly before removal. The withdrawal of the needle triggered a tail flick, followed by closure of the wound with sutures. Rats displaying signs of dyskinesia on the subsequent day were excluded from the study and euthanised by intraperitoneally injecting 100 mg/kg pentobarbital. Thoracotomy was performed on the remaining rats 21 days post-AAV administration.

### 2.5 Western blotting

Anterior cingulate cortex tissues were harvested and homogenized in radioimmunoprecipitation assay lysis buffer, with the addition of a protease and phosphatase inhibitor cocktail (CWBio, Beijing, China). The proteins extracted were subjected to electrophoresis on a 10% sodium dodecyl sulfate-polyacrylamide gel and then transferred to polyvinylidene fluoride (PVDF) membranes (CWBio). The PVDF membranes were first blocked with 5% non-fat milk at 23 °C for 2 h, followed by overnight incubation with primary antibodies at 4 °C. The membranes were then treated with horseradish peroxidase-conjugated anti-IgG antibody at 23 °C for 1 h (antibody details are provided in Table 1). An enhanced chemiluminescence kit (CWBio) was used to detect protein bands, and images were captured with the ImageQuant LAS4000 mini chemiluminescence imaging system (GE Healthcare, Chicago, IL, USA). ImageJ software (NIH) was utilized to perform data analysis.

**TABLE 1:** List of the primary and second antibodies used in the western blots.

Antibody	Host	Company	Catalog number	Dilution
Ror2	Rabbit	Cell signaling technology	88639	1:1000
CCL2	Rabbit	Abcam	Ab7202	1:2000
CXCL1	Goat	Invitrogen	PA5-46960	1:2000
GFAP	Rabbit	Proteintech	16825-1-AP	1:2000
C3	Rabbit	Abcam	ab200999	1:20
S100A10	Mouse	Cell signaling technology	5529	1:1000
β-actin	Mouse	Proteintech	66009-1-IG	1:2000
Anti-rabbit IgG HRP	Goat	Proteintech	SA00001-2	1:2000
Anti-mouse IgG HRP	Goat	Proteintech	SA00001-1	1:2000
Anti-goat IgG HRP	Donkey	Proteintech	SA00001-3	1:2000

### 2.6 Reverse transcription-polymerase chain reaction (RT-PCR)

RNA from the ACC was isolated using TRIzol reagent (15596026, Invitrogen, Carlsbad, CA, USA) with ultrasonic disruption. Prime Script RT Master Mix (RR036, Takara) was employed for reverse transcription. Supplementary Table 1 lists the primer sequences. For RT-PCR, SYBR Premix Ex Taq (RR820, Takara) was used, and amplification was performed on a Step One Real-Time PCR System (Applied Biosystems, Foster City, CA, USA).

### 2.7 Immunofluorescence

The rats were euthanised with an overdose of ketamine, followed by transcardial perfusion with phosphate-buffered saline, fixation in 4% paraformaldehyde for 12 h, and dehydration in 30% sucrose overnight at 4 °C. Tissues from the ACC were dissected and sectioned at a thickness of 20 μm using a Leica 2000 (Leica Microsystem, Germany). Bilateral ACC tissues were dissected according to the following coordinates: 3.20 mm anterior to −1.30 mm posterior to bregma, 0–0.8 mm lateral to midline and 1.2–3 mm vertical from brain surface. The slices underwent an initial 30 min incubation with 0.3% Triton-X-100, followed by blocking with 10% bovine serum albumin for 1 h. Primary antibodies were applied overnight at 4 °C, and secondary antibodies were incubated at 23 °C for 1 h (see Table 2 for details of the antibodies used). Immunofluorescent images were captured using a CCD spot camera (Olympus DP71, Masontec, Dublin, Ireland).

**TABLE 2:** List of the primary and secondary antibodies used for the immunofluorescence.

Antibody	Host	Company	Catalog number	Dilution
Ror2	Mouse	Santa cruz	sc-374174	1:200
CCL2	Rabbit	Abcam	Ab7202	1:50
CXCL1	Goat	Invitrogen	PA5-46960	1:20
GFAP	Chicken	Abcam	ab4674	1:500
C3	Rabbit	Abcam	ab11887	1:20
S100A10	Rabbit	Proteintech	11250-1-AP	1:200
Anti-chicken IgG Alexa Fluor 488	Goat	Abcam	ab150173	1:400
Anti-mouse IgG Alexa Flour 594	Donkey	Abcam	ab150108	1:400
Anti-rabbit IgG Alexa Flour 594	Donkey	Abcam	ab150076	1:400
Anti-goat IgG Alexa Fluor 594	Donkey	Abcam	ab150132	1:400

### 2.8 SCFA extraction and measurement

Short-chain fatty acids were analysed via gas chromatography-tandem mass spectrometry (GC-MS) by Metware Biotechnology Co., Ltd., (Wuhan, China). Fecal samples (20 mg) were prepared according to standard procedures. Analysis was conducted using an Agilent 7890B gas chromatograph paired with a 7000D mass spectrometer, fitted with a DB-FFAP column (30 m × 0.25 mm i.d. × 0.25 μm thickness, J&W Scientific, USA). The carrier gas was helium, with a flow rate of 1.2 mL/min. A 2 μL sample was injected in split mode. Starting at 90 °C for 1 min, the oven temperature ramped up to 100 °C at 25 °C/min, then to 150 °C at 20 °C/min, where it was maintained for 0.6 min. The temperature was increased to 200 °C at 25 °C/min and held for 0.5 min, completing a total run of 3 min. Samples were analysed in the multiple reaction monitoring mode. The injector temperature was kept at 200 °C, while the mass spectrometry transfer line was maintained at 230 °C.

### 2.9 Statistical analysis

The data are represented as the mean ± standard error of the mean. To analyse mechanical hyperalgesia and cold allodynia, two-way analysis of variance (ANOVA) was performed, and Bonferroni *post-hoc* tests were used for multiple comparisons. Biomolecular measurements were evaluated with one-way ANOVA, and the Student–Newman–Keuls test was used for *post-hoc* comparisons. For categorical data, results were reported as numbers and frequencies, with comparisons conducted using the chi-square test. GraphPad Prism (version 8.0, San Diego, CA, USA) was used for statistical analysis, and a *p*-value of <0.05 was deemed statistically significant.

## 3 Results

### 3.1 Post-thoracotomy hypersensitivity to mechanical and cold stimuli

**FIGURE 1:**
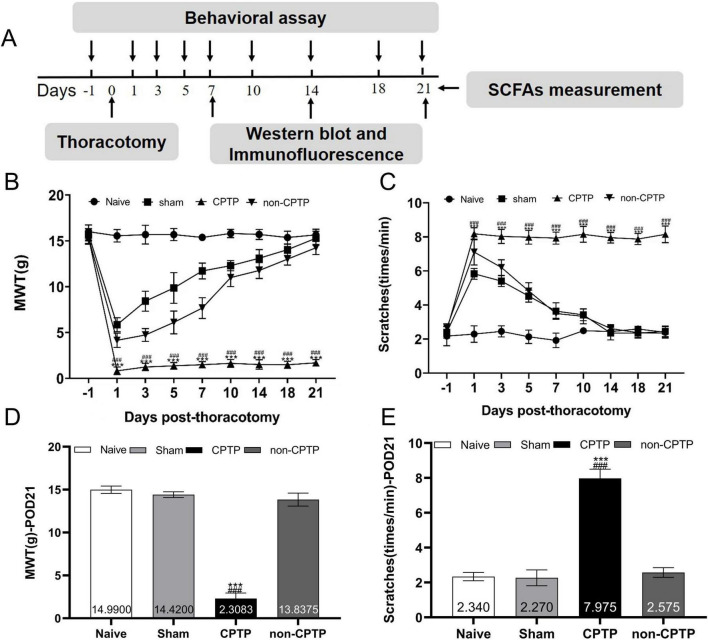
Modeling of CPTP in rats. **(A)** Overview of the experimental design. The procedures, including thoracotomy, behavioral tests, western blotting, immunofluorescence, and SCFAs measurement, were conducted at the scheduled time intervals. **(B)** Mechanical hyperalgesia induced by thoracotomy. **(C)** Cold allodynia induced by thoracotomy. **(D)** MWT histogram on POD 21 for all groups. **(E)** Scratching behavior histogram for all groups on POD 21. ^###^*P* < 0.001 compared with the non-CPTP group; ***P < 0.001 compared with the sham group; two-way ANOVA; group sizes: both sham and naïve (*n* = 10), non-CPTP (*n* = 13), and CPTP (*n* = 17).

Every procedure was carried out at the appointed times (Figure 1A). Rats were randomly grouped into three categories: model (*n* = 30), sham (*n* = 10), and naïve (*n* = 10). Within the model group, the rats were further categorized into non-CPTP and CPTP subgroups based on their MWT on postoperative day (POD) 21. Rats with an MWT ≤4 g on POD 21 were placed in the CPTP group, whereas those with a higher MWT were placed in the non-CPTP group. Not all patients who undergo thoracotomy in clinical practice develop CPTP. This variability renders the CPTP model distinct from other models. Pain prevalence in the model group aligned with previous research findings (Zhu et al., 2017, 2018). On POD 1, the MWT of the CPTP group decreased sharply and remained significantly low up to POD 21 (Figures 1B, D). Similarly, scratch counts increased on POD 1 and remained high up to POD 21 in CPTP rats (Figures 1C, E). On POD 1 and 7, rats in the non-CPTP and sham groups showed reduced MWT and higher scratch frequencies than those in the naïve group. From POD 10 to 21, there were no appreciable variations in MWT or scratch counts between the non-CPTP and sham groups (Figures 1B, C). These results confirm that thoracotomy induces CPTP in male rats.

### 3.2 CPTP upregulates Ror2, CCL2, and CXCL1 in the ACC

**FIGURE 2:**
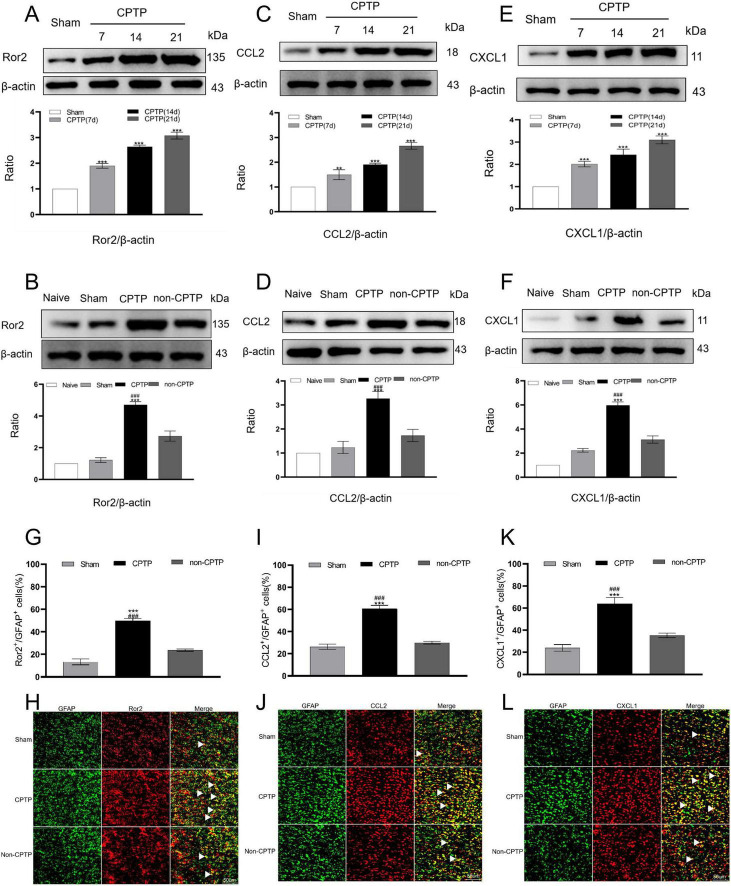
Upregulated levels of Ror2, CCL2, and CXCL1 in the ACC during CPTP. **(A,C,E)** Western blot analysis of Ror2, CCL2, and CXCL1 protein levels in the sham and CPTP groups on POD 7, 14, and 21. **(B,D,F)** Western blot analysis of Ror2, CCL2, and CXCL1 protein levels on POD 21. **(G,I,K)** Quantitative analysis of Ror2, CCL2, or CXCL1 co-localization with GFAP on POD 21. **(H,J,L)** On POD 21, immunofluorescence analysis showed the co-localization of Ror2, CCL2, and CXCL1 (all red) with GFAP (green). Scale bar: 50 μm; ^###^*P* < 0.001 compared with the non-CPTP group; **P < 0.01, and ***P < 0.001 compared with the sham group; one-way ANOVA; *n* = 3 per group.

The CPTP group exhibited significantly higher Ror2 protein expression in the ACC from POD 7 to 21 than the sham group (Figure 2A). Moreover, on POD 21, the CPTP group showed a pronounced elevation in Ror2 protein levels when compared to the non-CPTP, sham, and naïve groups (Figure 2B). Double immunofluorescence staining demonstrated that Ror2 was more prominently co-localized with glial fibrillary acidic protein (GFAP) in the CPTP group than in the non-CPTP and sham groups (Figures 2G, H).

On POD 7, 14, and 21, the CPTP group demonstrated significantly elevated astrocytic CCL2 and CXCL1 levels in the ACC when compared to levels in the sham group (Figures 2C, E). Furthermore, the CPTP group showed noticeably greater levels of CCL2 and CXCL1 on POD 21 than the other groups (Figures 2D, F). Double immunofluorescence staining further confirmed that the CPTP group exhibited greater co-localization of CCL2 and CXCL1 with GFAP than the other groups (Figures 2I, J, K, L).

### 3.3 CPTP induced A1 phenotype polarization in the ACC

**FIGURE 3:**
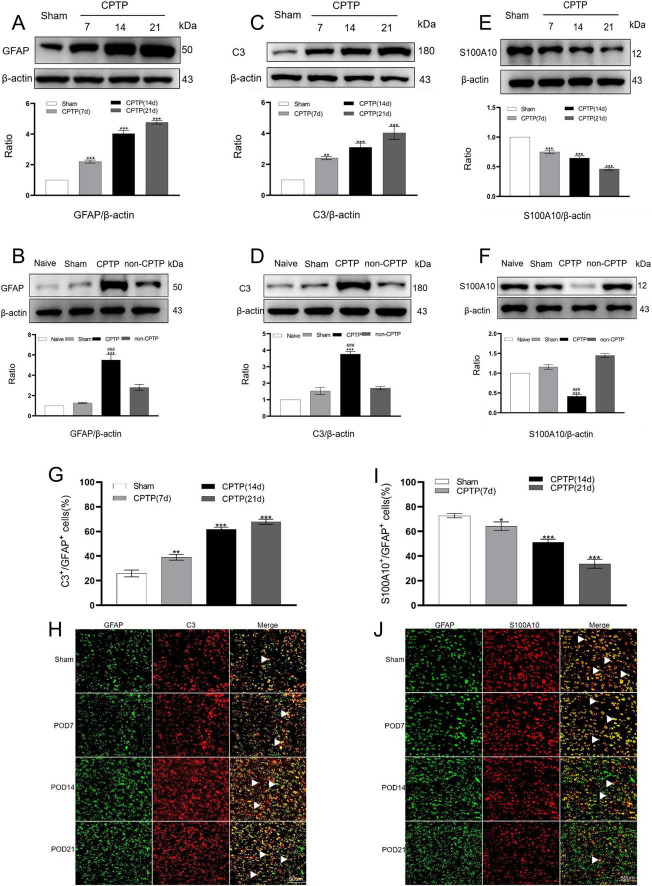
CPTP primarily drove the transition of astrocytes in the ACC to the A1 phenotype. **(A,C,E)** Western blotting of GFAP, C3, and S100A10 levels in the CPTP and sham groups across POD 7, 14, and 21. **(B,D,F)** Western blotting of GFAP, C3, and S100A10 levels in all groups on POD 21. **(G,I)** Quantitative analysis of C3 or S100A10 co-localization with GFAP on POD 7, 14, and 21. **(H,J)** Immunofluorescence analysis revealed C3 or S100A10 (all red) co-localization with GFAP (green) on POD 7, 14, and 21. Scale bar: 50 μm. ^###^*P* < 0.001 compared with the non-CPTP group; *P < 0.05, **P < 0.01, and ***P < 0.001 compared with the sham group; one-way ANOVA; *n* = 3 per group.

In the sham group, protein levels were determined by averaging the values from all sham group samples (POD 7, 14, and 21). GFAP levels in the CPTP group were significantly higher from POD 7 to 21 than those in the sham group (Figure 3A). C3 expression, a marker of the A1 phenotype, was elevated on POD 7, 14, and 21, compared with that in the sham group (Figure 3C). Conversely, the levels of S100A10 (a marker of the A2 phenotype) were reduced at these time points post-thoracotomy (Figure 3E). On POD 21, the CPTP group had higher levels of GFAP and C3 protein than the non-CPTP, sham, and naïve groups (Figures 3B, D), but S100A10 levels were lower (Figure 3F).

Double immunofluorescence staining to further investigate the distribution of A1 and A2 astrocytes in the ACC revealed that C3 and S100A10 were primarily co-localized with GFAP in CPTP rats (Figures 3H, J). Over time, the A1 phenotype exhibited an increase in comparison with that in the sham group, while the A2 phenotype declined in the CPTP group (Figures 3G, I). These findings suggest that in the rats with CPTP, reactive astrocytes in the ACC predominantly exhibit the A1 phenotype, with a weak A2 phenotype, particularly during the chronic stage.

### 3.4 Astrocytic Ror2 knockdown alleviates thoracotomy-induced pain

To investigate how elevated ACC Ror2 expression contributes to pain behavior and astrocytic polarization during CPTP, rats were pre-treated with spinal AAV2/9-GFAP-miR30-shRor2 before thoracotomy. The procedure was carried out at the appointed times (Supplementary Figure 1A). The effectiveness of intrathecal AAV administration in knocking down protein expression in the brain was demonstrated in our previous study (Zhu et al., 2023). Ror2 downregulation by AAV2/9-GFAP-miR30-shRor2 was verified through western blotting and RT-PCR (Supplementary Figures 1B, C). Notably, astrocyte-specific Ror2 knockdown did not affect the baseline pain threshold (Supplementary Figures 1D, E), highlighting the therapeutic potential of this targeted approach for selective modulation without affecting normal nociceptive processing.

**FIGURE 4:**
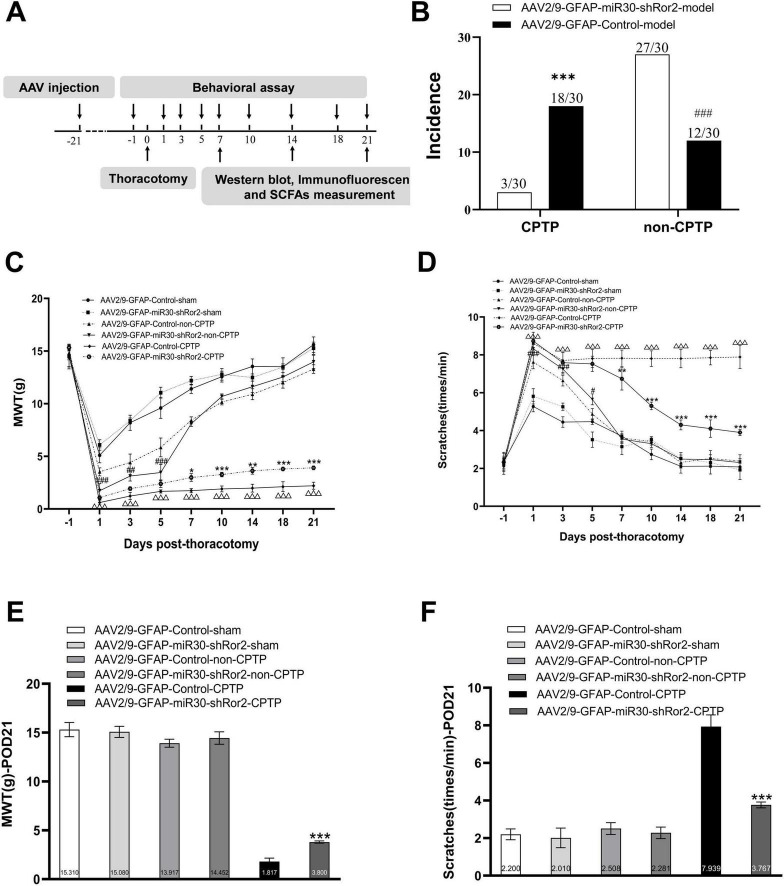
Astrocytic Ror2 knockdown mitigated pain behaviors associated with thoracotomy. **(A)** Design overview of the experiment. Intrathecal injection of AAV2/9-GFAP-miR30-shRor2 (40 μL) and AAV2/9-GFAP-Control (40 μL) 21 days before thoracotomy. Pain behavioral testing, western blotting, immunofluorescence, and SCFAs measurement were conducted at specified intervals. **(B)** The incidence rates of CPTP and non-CPTP were compared between the AAV2/9-GFAP-miR30-shRor2 and AAV2/9-GFAP-Control groups on POD 21. ^#^*P* < 0.05, ^##^*P* < 0.01, and ^###^*P* < 0.001 compared with the AAV2/9-GFAP-Control-non-CPTP group; *P < 0.05, **P < 0.01, and ***P < 0.001 compared with the AAV2/9-GFAP-Control-CPTP group; using chi-square test. **(C)** Evaluation of mechanical hyperalgesia in CPTP and non-CPTP rats following intrathecal injection of AAV2/9-GFAP-miR30-shRor2. **(D)** Evaluation of cold allodynia by scratching behaviors following intrathecal AAV2/9-GFAP-miR30-shRor2 injection. **(E)** MWT histogram on POD 21 for all groups. **(F)** Scratching behavior histogram for all groups on POD 21. *P < 0.05, **P < 0.01, and ***P < 0.001 compared with the AAV2/9-GFAP-Control-CPTP group; ^#^*P* < 0.05, ^##^*P* < 0.01, and ^###^*P* < 0.001 compared with the AAV2/9-GFAP-Control-non-CPTP group; ^△^*P* < 0.05, ^△△^*P* < 0.01, ^△△△^*P* < 0.001 compared with the AAV2/9-GFAP-Control-sham; two-way ANOVA; group sizes: both AAV2/9-GFAP-miR30-shRor2-sham and AAV2/9-GFAP-Control-sham (*n* = 10), AAV2/9-GFAP-Control-non-CPTP (*n* = 12), AAV2/9-GFAP-Control-CPTP (*n* = 18), AAV2/9-GFAP-miR30-shRor2-non-CPTP (*n* = 27), and AAV2/9-GFAP-miR30-shRor2-CPTP (*n* = 3).

To evaluate the impact on pain modulation, an intrathecal injection of AAV2/9-GFAP-miR30-shRor2 (40 μL) or its control vector AAV2/9-GFAP-Control (40 μL) was performed 21 days before thoracotomy (Figure 4A). Rats were randomly assigned to one of four primary groups: AAV2/9-GFAP-Control-sham (*n* = 10), AAV2/9-GFAP-Control model (*n* = 30), AAV2/9-GFAP-miR30-shRor2-sham (*n* = 10), and AAV2/9-GFAP-miR30-shRor2 model (*n* = 30). Following the surgery, the rats in the two “model” groups were further subdivided based on their MWT on POD 21. This stratification resulted in the following subgroups for analysis: AAV2/9-GFAP-Control-non-CPTP, AAV2/9-GFAP-Control-CPTP, AAV2/9-GFAP-miR30-shRor2-non-CPTP, and AAV2/9-GFAP-miR30-shRor2-CPTP. Early AAV2/9-GFAP-miR30-shRor2 treatment effectively alleviated thoracotomy-induced chronic pain, demonstrating its potential in preventive pain management. The intervention’s potential effectiveness was highlighted by the substantial reduction in the incidence of CPTP in the AAV2/9-GFAP-miR30-shRor2 group, which decreased from 18/30 in the AAV2/9-GFAP-Control group to 3/30 (Figure 4B). In addition, from POD 1 to 5, the MWT of the AAV2/9-GFAP-miR30-shRor2-non-CPTP group showed a sustained decrease compared to that of the AAV2/9-GFAP-Control-non-CPTP group (Figure 4C). From POD 7 to 21, the MWT in the AAV2/9-GFAP-miR30-shRor2-CPTP group was significantly higher than that in the AAV2/9-GFAP-Control-CPTP group (Figures 4C, E), suggesting long-term modulation of pain sensitivity.

In contrast to the AAV2/9-GFAP-Control-non-CPTP group, the AAV2/9-GFAP-miR30-shRor2-non-CPTP group showed a marked increase in scratching behavior from POD 1 to 5 (Figure 4D). Conversely, the AAV2/9-GFAP-miR30-shRor2-CPTP group demonstrated a substantial and sustained reduction in scratching from POD 7 to 21, compared with that in the AAV2/9-GFAP-Control-CPTP group (Figures 4D, F). The findings revealed that AAV2/9-GFAP-miR30-shRor2 treatment resulted in elevated MWT levels and a notable decrease in cold acetone-induced scratching in CPTP rats across POD 7 to 21, suggesting that suppressing astrocytic Ror2 in the ACC effectively mitigates pain behaviors associated with CPTP.

### 3.5 Astrocytic Ror2 knockdown restores the A1/A2 ratio imbalance in the ACC of CPTP rats

**FIGURE 5:**
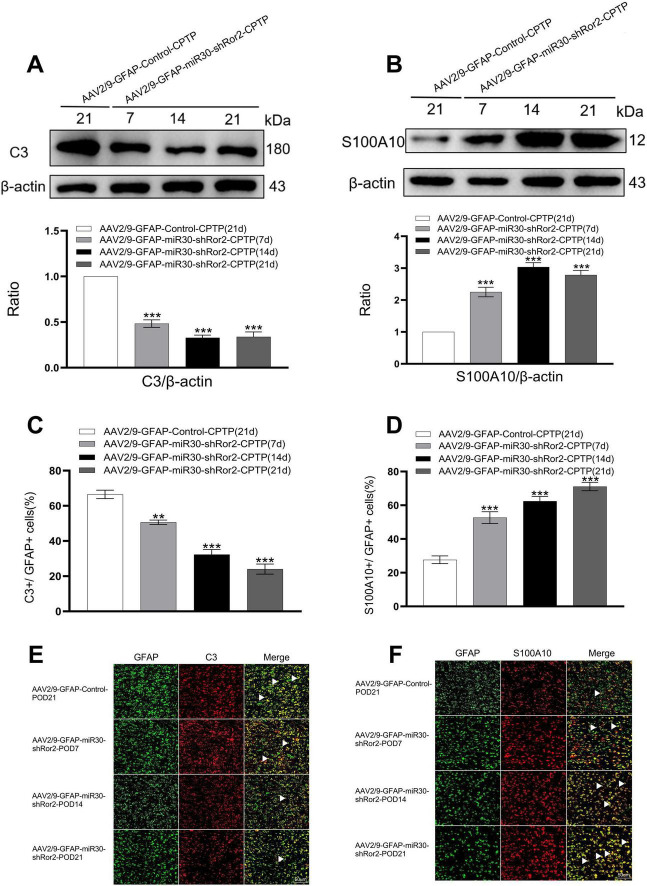
Astrocytic Ror2 knockdown rebalanced the A1/A2 ratio in the ACC in CPTP rats. **(A,B)** Western blotting analysis of C3 and S100A10 after spinal delivery of AAV2/9-GFAP-miR30-shRor2 or AAV2/9-GFAP-Control in CPTP rats. **(C,D)** Quantitative co-localization analysis of C3 or S100A10 with GFAP in the AAV2/9-GFAP-miR30-shRor2-CPTP group compared with the AAV2/9-GFAP-Control-CPTP group (POD 21), *n* = 3 per group. **P < 0.01, and ***P < 0.001 compared with the AAV2/9-GFAP-Control-CPTP group; one-way ANOVA; *n* = 3 per group. **(E,F)** Immunofluorescence staining revealed co-localization of C3 or S100A10 (all red) with GFAP (green) in the AAV2/9-GFAP-miR30-shRor2-CPTP group on POD 7, 14, and 21 compared with the AAV2/9-GFAP-Control-CPTP group on POD 21; *n* = 3 per group.

To determine the effects of astrocytic Ror2 knockdown on ACC astrocytes polarization during CPTP, two phenotypic markers, C3 and S100A10, were co-localized with reactive astrocyte GFAP in the AAV2/9-GFAP-miR30-shRor2-CPTP and AAV2/9-GFAP-Control-CPTP groups. Our initial study identified POD 21 as the peak of reactive astrocytes in CPTP rats. Thus, POD 21 in the AAV2/9-GFAP-Control-CPTP group was used as a control. In the AAV2/9-GFAP-miR30-shRor2-CPTP group, decreased C3 expression and elevated S100A10 expression were observed on POD 7, 14, and 21, as revealed by western blot analysis (Figures 5A, B).

Double immunofluorescence revealed a phenotypic transition in astrocytes, with reduced A1 and enhanced A2 phenotypes in the AAV2/9-GFAP-miR30-shRor2-CPTP group compared with those in the AAV2/9-GFAP-Control-CPTP group (white arrow in Figures 5E, F). Histograms further revealed that C3 or S100A10 co-localized with GFAP (Figures 5C, D), suggesting that astrocytic Ror2 knockdown modulates the reactive astrocytic phenotype by reestablishing the A1/A2 balance in CPTP rats.

### 3.6 Astrocytic Ror2 knockdown downregulates CCL2 and CXCL1 expression in the ACC of CPTP rats

**FIGURE 6:**
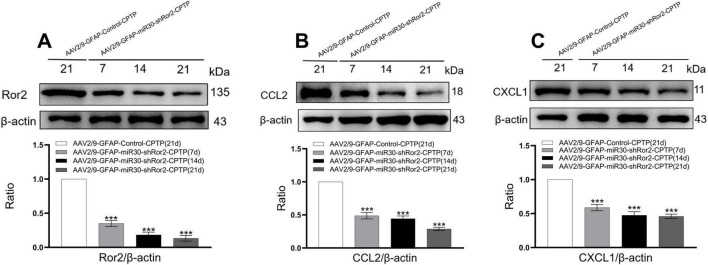
Astrocytic Ror2 knockdown downregulated CCL2 and CXCL1 in the ACC in CPTP rats. **(A,B,C)** Western blotting of Ror2, CCL2, and CXCL1 levels on POD 7, 14, and 21. ***P < 0.001 compared with the AAV2/9-GFAP-Control-CPTP group (POD 21); one-way ANOVA; *n* = 3 per group.

To evaluate the expression of Ror2, CCL2, and CXCL1 in the ACC, western blotting was performed on CPTP rats following spinal AAV2/9-GFAP-miR30-shRor2 administration. On POD 7, 14, and 21, Ror2 expression in the ACC of the AAV2/9-GFAP-miR30-shRor2-CPTP group was significantly decreased compared with that in the AAV2/9-GFAP-Control-CPTP group (Figure 6A). Additionally, the AAV2/9-GFAP-miR30-shRor2-CPTP group exhibited significantly lower levels of CCL2 (Figure 6B) and CXCL1 (Figure 6C). These findings suggest that early intrathecal injection of AAV2/9-GFAP-miR30-shRor2 effectively mitigates the CPTP-induced elevation in Ror2, CCL2, and CXCL1 levels in the ACC.

### 3.7 Astrocytic Ror2 knockdown restores SCFAs levels in CPTP rats

**FIGURE 7:**
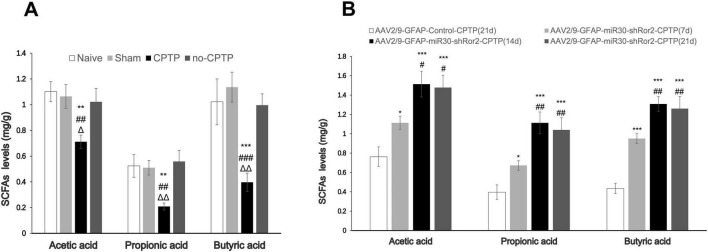
Spinal delivery of AAV2/9-GFAP-miR30-shRor2 restored SCFA levels in CPTP rats. **(A)** GC-MS analysis showing the SCFA levels in guts of the naïve, sham, CPTP, and non-CPTP groups on POD 21. ^Δ^*P* < 0.05 and ^ΔΔ^*P* < 0.01 compared with the non-CPTP group; ^##^*P* < 0.01 and ^###^*P* < 0.001 compared with the sham group; **P < 0.01 and ***P < 0.001 compared with the naïve group; one-way ANOVA; *n* = 6 per group. **(B)** GC-MS analysis showing the SCFA levels in the guts of the AAV2/9-GFAP-miR30-shRor2-CPTP group on POD 7, 14, and 21 compared with the AAV2/9-GFAP-Control-CPTP group on POD 21. ^#^*P* < 0.05 and^##^*P* < 0.01 compared with the AAV2/9-GFAP-miR30-shRor2-CPTP group (POD 7); *P < 0.05, **P < 0.01, and ***P < 0.001 compared with the AAV2/9-GFAP-Control-CPTP group; one-way ANOVA; *n* = 6 per group.

Compared with those in the non-CPTP, sham, and naïve groups, the CPTP group showed a noticeable reduction in SCFA levels on POD 21 (Figure 7A). On POD 7, 14, and 21, SCFA levels in the AAV2/9-GFAP-miR30-shRor2-CPTP group were significantly elevated compared to those in the AAV2/9-GFAP-Control-CPTP group on POD 21 (Figure 7B). However, in the AAV2/9-GFAP-miR30-shRor2-CPTP group, SCFA production showed no significant difference between POD 14 and 21 (Figure 7B). These results indicate that reduced SCFAs in the gut may contribute to CPTP pathogenesis and that Ror2 suppression in astrocytes restored SCFA levels in CPTP rats.

## 4 Discussion

Based on the rat rib-retraction model of CPTP developed by Buvanendran et al. (2004), we observed upregulation of Ror2, CCL2, and CXCL1 in ACC astrocytes. Reactive astrocytes in the ACC predominantly exhibited the A1 phenotype, and SCFA production decreased in CPTP rats. The reduced expression of Ror2 following intrathecal AAV2/9-GFAP-miR30-shRor2 injection effectively alleviated pain behaviors, lowered astrocytic CCL2 and CXCL1 levels, reversed the A1/A2 astrocyte ratio, and restored SCFA levels. These findings highlight the role of astrocytic Ror2 in regulating A1/A2 phenotypic transition, secreting CCL2 and CXCL1 in the ACC, and improving SCFA levels in the gut microbiota during CPTP. The results support our initial hypothesis that early intervention targeting astrocyte phenotypes in the ACC may affect the brain–gut axis, ultimately enhancing metabolite production.

Astrocytes are progressively being identified as significant contributors to chronic pain pathogenesis (Li et al., 2019). The ACC, which is the crucial medial pathway of pain, shows heightened activity in response to neuropathic pain, as demonstrated by neuroimaging studies (Peyron et al., 2000). Reactive astrocytes in this area is a prominent feature in neuropathic pain models (Xu et al., 2008; Yamashita et al., 2014). Similarly, our study revealed elevated GFAP expression in the ACC of CPTP rats, suggesting the involvement of activated astrocytes in CTPT development. Recently, reactive astrocytes have been categorized into A1 and A2, and their potential roles in neuroscience have been elucidated (Liddelow et al., 2017). Previously, it was reported that reactive astrocytes in the spinal cord primarily adopt the A1 phenotype during CPTP progression and that suppressing the polarization of A1 astrocytes inhibits chronic pain (Liu et al., 2022). In this study, the A1 phenotype was predominant in the ACC during CPTP, with few A2 subtypes observed. Therefore, our findings suggest that the A1/A2 astrocytes transition in the ACC contributes to CPTP pathogenesis.

Neuroinflammation, characterized by the activation of astrocytes and microglia in the central nervous system along with chemokine release, initiates central sensitisation. The chemokines CCL2 and CXCL1 have been widely studied in chronic pain. Abbadie et al. (2003) documented that sciatic nerve ligation elevated CCL2 expression in both the spinal cord and primary sensory neurons, with CCL2-positive spinal cells identified as astrocytes. The upregulation of CXCL1 at both the protein and mRNA levels in spinal astrocytes suggests their participation in neuropathic pain development (Zhang et al., 2013). In addition to the spinal cord, CCL2 and CXCL1 are also involved in neuronal injury in the brain and spinal nerve ligation (Guo et al., 2012; Katayama et al., 2009). Our findings demonstrated increased CCL2 and CXCL1 expression in the ACC astrocytes during CPTP, suggesting that these chemokines, released by reactive astrocytes, contribute to chronic pain development.

Orphan receptor 2, the receptor for Wnt5a, is involved in neuronal development and plasticity (Petrova et al., 2014). Zhou et al. (2019) observed spinal Ror2 upregulation in mice with chronic constriction injury (CCI) and found that specific Ror2 knockdown effectively prevented CCI-induced pain behaviors. Our recent study revealed that Wnt5a inhibition significantly increased the pain threshold and decreased Ror2 level in CPTP rats (Zhu et al., 2018). Similarly, in the same model, Ror2 expression in the ACC was elevated, while Ror2 knockdown resulted in a reduction in allodynia. Based on these findings, it is suggested that Ror2 upregulation in the ACC may contribute to the development of CPTP. Notably, Ror2 knockdown decreased the prevalence and severity of chronic pain. However, the rats experienced pain in the early stages following thoracotomy, indicating that Ror2 may not have a significant impact on acute pain. This implies that other mechanisms contribute to the occurrence of acute postoperative pain. For the cyto-location of Ror2, astrocytic Ror2 levels are upregulated after brain injury, and specific knockdown of Ror2 in astrocytes leads to a marked decrease in astrocyte proliferation and density (Endo et al., 2017). In this study, we also observed that Ror2 strongly co-localized with astrocytes. The downregulation of astrocyte-specific Ror2 has been linked to changing astrocyte phenotypes, specifically reversing the A1/A2 imbalance in the ACC in the context of CPTP progression. Furthermore, reduced CCL2 and CXCL1 expression was observed in astrocytes following astrocytic Ror2 knockdown. Ror2 potentially contributes to the reactive astrocytes and promotes CCL2 and CXCL1 synthesis in the ACC. Further studies are essential to define the exact role of Ror2 in regulating astrocyte reactivity and phenotype transitions in the ACC.

Short-chain fatty acids exert beneficial effects on different pain types (Bonomo et al., 2020; Russo et al., 2016). Butyrate, the most extensively studied SCFA, has been shown to reduce neuropathic pain in obese mice (Bonomo et al., 2020). Moreover, oral butyrate alleviates visceral and neuropathic pain (Russo et al., 2016). There is less research on how propionate and acetate affect pain modulation compared to studies on butyrate. A study indicated that patients with fibromyalgia had significantly reduced levels of propionate (Minerbi et al., 2019). In a mouse model with migraine-like pain triggered by nitroglycerin, sodium propionate administration resulted in pain relief (Lanza et al., 2021). However, evidence for the role of acetate in pain regulation is lacking. Contrary to the above studies, antibiotic treatment alleviated neuropathic pain in a CCI model; however, this effect was blocked by SCFA administration (Zhou et al., 2021). The gut–brain axis, with its bidirectional regulation, may create a feedback loop in which alterations in the brain influence gut function. Wu et al. (2023) reported that TREM-1 and TREM-2, which are innate immune receptors on microglia in the ACC, contribute to visceral hypersensitivity in a dextran sulfate sodium-induced colitis model. Our study demonstrated that the reduced levels of SCFAs in the CPTP model were reversed when pain was relieved by Ror2 knockdown, suggesting that SCFAs may improve CPTP. Further investigations are necessary to determine if exogenous SCFAs could help alleviate CPTP.

There are two limitations in the design of this study. After intrathecal AAV injection, we noted a reduction in Ror2 expression in the ACC, suggesting that AAV can access the brain through cerebrospinal fluid circulation. However, reactive astrocytes in the spinal cord may affect brain function via immunomodulatory mechanisms. Furthermore, our study mainly examined the effects of intrathecal injections on the brain and gut, without exploring the potential influence of gut microbiota metabolites on central nervous system function, which requires further investigation.

## 5 Conclusion

Our study highlights the important role of astrocytic Ror2 in regulating the polarization of astrocytes, synthesis of CCL2 and CXCL1 in the ACC, and levels of gut SCFAs during the initiation and maintenance of CPTP. Inhibiting astrocytic Ror2 in the ACC may be a new therapeutic method for preventing and alleviating CPTP.

## Data Availability

The original contributions presented in the study are included in the article/Supplementary material, further inquiries can be directed to the corresponding author/s.
