# Supplementary material for: Astrocytic Ror2-induced imbalance in brain and gut homeostasis contributes to chronic post-thoracotomy pain
Source: Front Aging Neurosci. 2025 Nov 7;17:1675725. doi: 10.3389/fnagi.2025.1675725 (PMC12634580; doi:10.3389/fnagi.2025.1675725)
Supplement: Supplementary file 1 [file Table_1.docx]

**
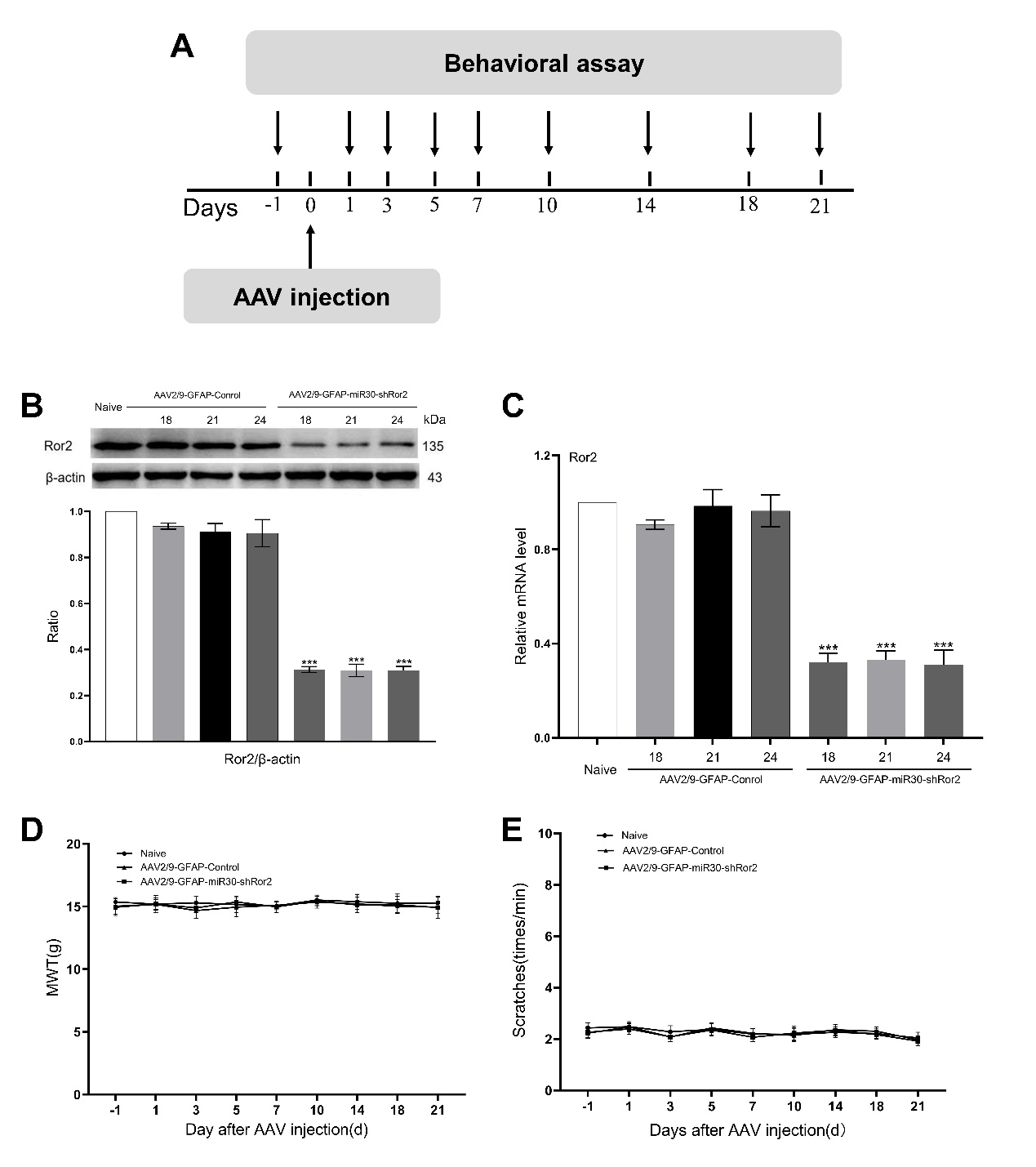
**

**Fig. S1. Assessment of the effectiveness of AAV injection.** (**A**) Schematic diagram of the experimental design. AAV2/9-GFAP-miR30-shRor2 (40 μL) and AAV2/9-GFAP-Control (40 μL) were injected intrathecally into naïve rats. Pain behavioral assessments were conducted at the specified time intervals. (**B**) Western blot analysis of Ror2 protein expression levels in the ACC following intrathecal injection of AAV2/9-GFAP-miR30-shRor2 or AAV2/9-GFAP-Control at the specified time points. The expression of Ror2 was normalized to that of β-actin in each sample, with Ror2 level in the naïve group set as 1 for quantification. (**C**) RT-PCR analysis of Ror2 mRNA expression levels in the ACC of naïve rats following intrathecal injection of AAV2/9-GFAP-miR30-shRor2 or AAV2/9-GFAP-Control at the specified time points. The expression of Ror2 was normalized to that of GAPDH in each sample, with Ror2 level in the naïve group set as 1 for quantification. Statistical analysis was conducted using one-way ANOVA, n = 3 per group. (**D**) MWT of the right back in response to the von Frey filaments at the specified time points. Statistical analysis was conducted using two-way ANOVA, n = 10 per group. (**E**) Cold allodynia of the right back at the specified time points. Statistical analysis was conducted using two-way ANOVA, n = 10 per group. ** P* < 0.05, *** P* < 0.01, and **** P* < 0.001 when compared with the naïve group.

Supplementary Table 1 List of the primer sequences for RT-PCR.

| Target | |  | Primer sequence 5′-3′ | Product length(bp) | GenBank NO. |
| --- | --- | --- | --- | --- | --- |
| Ror2 | FORWARD | | TGGGAACCGAACTATTTATGTG | 154 | NM_001107339 |
|  | REVERSE | | AGGAAAGACGAAGTGGCAGA |  |  |
